# An arrangement of secretory cells involved in the formation and storage of resin in tracheid-based secondary xylem of arborescent plants

**DOI:** 10.3389/fpls.2023.1268643

**Published:** 2023-09-05

**Authors:** Mirela Tulik, Joanna Jura-Morawiec

**Affiliations:** ^1^Department of Forest Botany, Institute of Forest Sciences, Warsaw University of Life Sciences, Warsaw, Poland; ^2^Polish Academy of Sciences Botanical Garden - Centre for Biological Diversity Conservation in Powsin, Warsaw, Poland

**Keywords:** resin, secretory cells, ducts, conifers, *Dracaena* spp., defense mechanism

## Abstract

The evolution of the vascular system has led to the formation of conducting and supporting elements and those that are involved in the mechanisms of storage and defense against the influence of biotic and abiotic factors. In the case of the latter, the general evolutionary trend was probably related to a change in their arrangement, i.e. from cells scattered throughout the tissue to cells organized into ducts or cavities. These cells, regardless of whether they occur alone or in a cellular structure, are an important defense element of trees, having the ability to synthesize, among others, natural resins. In the tracheid-based secondary xylem of gymnosperms, the resin ducts, which consist of secretory cells, are of two types: axial, interspersed between the tracheids, and radial, carried in some rays. They are interconnected and form a continuous system. On the other hand, in the tracheid-based secondary xylem of monocotyledons, the resin-producing secretory cells do not form specialized structures. This review summarizes knowledge on the morpho-anatomical features of various types of resin-releasing secretory cells in relation to their: (i) location, (ii) origin, (iii) mechanism of formation, (iv) and ecological significance.

## 1 Introduction

Various types of secondary growth have emerged during evolution. A special type of secondary growth has appeared in some monocotyledons, which is a manifestation of the activity of the monocot cambium producing secondary xylem along with secondary phloem in the form of vascular bundles (Carlquist, 2012; Jura-Morawiec et al., 2015; Maděra et al., 2020; Jura-Morawiec et al., 2021; Tulik et al., 2022). Another product of the monocot cambium is the parenchyma, the cells of which fill the space between the vascular bundles and constitute a large part of the secondary growth (Hubálková et al., 2017). The secondary xylem in monocotyledons is represented by tracheids (Carlquist, 2012; Jura-Morawiec, 2017). The secondary xylem in conifers is formed from the vascular cambium and includes both tracheids and parenchyma cells. In addition, the secondary xylem is spatially separated from the secondary phloem and contains only about 10% of parenchyma cells (Evert, 2006). Xylem parenchyma cells perform many functions, i.e. they participate in the transport and storage of water (Johnson et al., 2012; Klein et al., 2016; Carlquist, 2018), are a good neighbor and take part in postmortem tracheary element walls lignification (Baghdady et al., 2006; Smith et al., 2013; Blokhina et al., 2019), they re-fill cavitated tracheary elements (Spicer, 2014; Secchi et al., 2017), affect the mechanical properties of xylem (Reiterer et al., 2002; Arbellay et al., 2012), accumulate reserve substances (Tomasella et al., 2019; Słupianek et al., 2021), synthesize secondary metabolites in the process of heartwood formation (Hillis, 1987), participate in compartmentalization after tree injury (Morris et al., 2016), and secrete resin (Back, 2002; Cabrita, 2019). The latter function is performed by specialized parenchyma cells forming secretory structures (Bannan, 1936; Nagy et al., 2000). Among the monocotyledons with tracheid- based secondary xylem, there is only a small group of plants in the genus Dracaena, known as dragon trees, which have the ability to secrete resin (Maděra et al., 2020; Liu et al., 2021), which have the ability to secrete resin. Unlike conifers, in the secondary growth of dragon tree, this function is performed by single parenchyma cells (Jura-Morawiec and Tulik, 2015). However, these cells are difficult to identify among other parenchyma cells and it is not known whether this is their only function or whether they have many functions.

Although the secondary xylem of both conifers and dragon trees is based on tracheids, the parenchyma cells present in their bodies differ in terms of resin synthesize and secretion processes, therefore in this review we summarize the knowledge on the morpho-anatomical features of various types of resin-releasing secretory cells, taking into account their: (i) location, (ii) origin, (iii) mechanism of formation, (iv) and ecological significance.

## 2 Resin ducts in the secondary xylem of coniferous trees

In conifers such as *Cathaya*, *Pinus*, *Picea*, *Larix*, *Pseudotsuga*, and *Keteleeria* (*K. davidiana*, *K. evelyniana*) resin ducts (syn. resin canals) are a normal feature of the secondary xylem, and their formation can also be induced by external factors leading to traumatic resin duct development. In contrast, *Abies*, *Nothotsuga*, *Tsuga*, *Cedrus* or *Pseudolarix* are capable of producing only traumatic resin ducts in the secondary xylem (Bannan, 1936; Wu and Hu, 1997; Hudgins et al., 2005; Arbellay et al., 2014; Esteban et al., 2021).

Among the various criteria for distinguishing the secretory structures involved in resin synthesis, secretion, and accumulation under hydrostatic pressure, one is based on their anatomical structure. In the secondary xylem of *Abies*, *Cedrus*, *Tsuga*, and *Pseudolarix*, the resin-producing cells form blisters, which are a sac-like structure. This structure is surrounded with a layer of parenchyma cells termed epithelial cells (Wu and Hu, 1997). These cells die in the short time and their walls are lignified. In turn, in *Cathaya*, *Pinus*, *Picea*, *Larix* and *Pseudotsuga* the tube-like resin ducts are found. In these genera, thick-walled (except *Pinus*, which has stretchable, thin-walled epithelial cells) and long-lived secretory epithelial cells synthesize resin (Nagy et al., 2000). Not only the thickness of the epithelial cell walls varies, but also their number depending on the conifers. i.e. in *Pinus sylvestris* there are usually 4-6 cells surrounding the lumen of the duct while in *Picea, Larix* or *Pseudotsuga* there are 7-12 cells around the lumen (Schweingruber, 1978). In addition, the epithelium may be surrounded by 1-3 layers of pectin-enrich subsidiary cells easily distinguishable morpho-anatomically from epithelial cells and crushed during the development of the duct (Wiedenhoeft and Miller, 2002; Esteban et al., 2005). The elongate crystals both in epithelial and subsidiary parenchyma cells may also be present (Wiedenhoeft et al., 2003).

Resin ducts are classified to their arrangement as axial and radial. Epithelial cells of the axial resin ducts originate from fusiform cambial initials, while those of the radial resin ducts originate from ray cambial initials. The lumen between the epithelial cells can be formed by cells separation (schizogenous), cell lysis (lysigenous), or through a developmental process that involves both schizogenous and lysigenous pathways, known as schizolysigenous (Turner, 1999). The most commonly described lumen duct formation is that of schizogenous formation (Nagy et al., 2000) and occurs where it forms between the initial of the epithelial cells after hydrolysis of the middle lamella that binds the cells together. Auxin, which is involved in the secondary xylem formation, may promote the differentiation of resin ducts (Aloni, 2021). Since resin ducts do not form until several weeks after auxin application, hence it is assumed that in conifers the effect of auxin on resin duct development may include auxin-ethylene crosstalk (Fahn, 1988; Hudgins et al., 2006; Arbellay et al., 2014).

**Figure 1:**
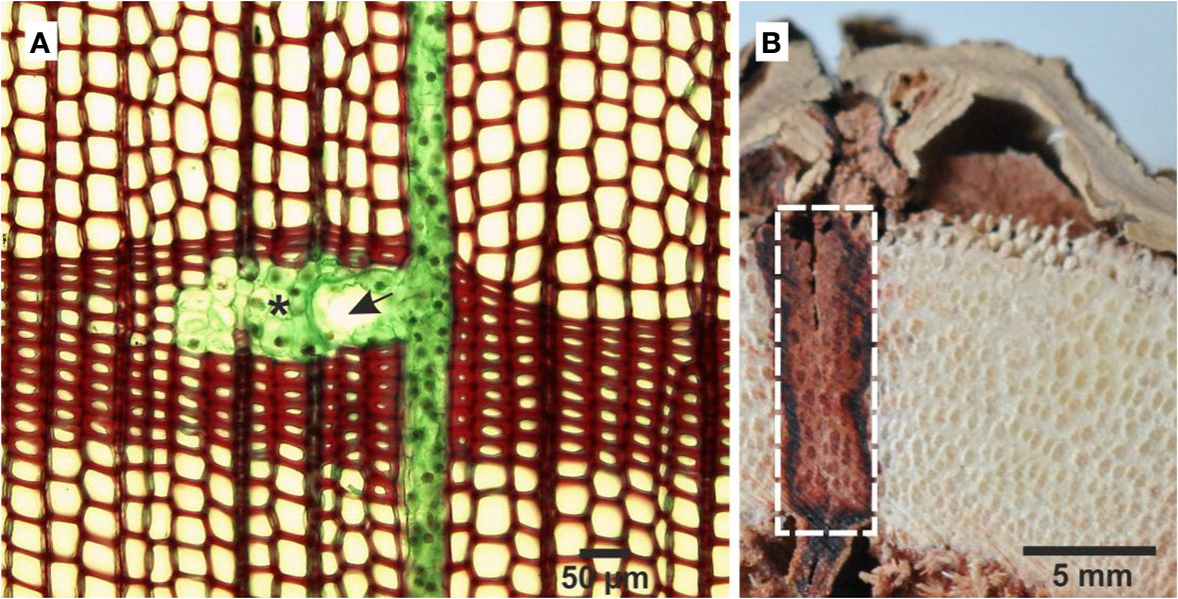
Cross sections through the trunk of *Pinus sylvestris* **(A)** and *Dracaena draco* **(B)**. The axial resin duct and the ray carrying the radial resin duct (fusiform ray), which together form a co-planar network in the secondary xylem. The asterisk denotes subsidiary cell, and the arrow denotes epithelial cell with thin, cellulose cell walls **(A)**. The white-sided rectangle covers the wound area with visible dragon’s blood in the tracheids of the vascular bundles and in the parenchyma cells of the secondary growth **(B)**.

Resin ducts form co-planar networks in conifers secondary xylem (**Figure 1A**). Axial resin ducts, with an average diameter of 200 µm, are usually found in the outer region of the earlywood and in the first-formed latewood in every annual ring. Under normal conditions there are only a few, scattered axial resin ducts in the secondary xylem (Fahn et al., 1979; Wu and Hu, 1997). In spruce they usually occur singly or in pairs, rarely in groups of three in close proximity to each other. Normally, the axial resin ducts become longer as the age of the cambium increases (LaPascha and Wheeler, 1990; Krokene et al., 2008). Radial resin ducts start with vertical ducts and appear in some rays. Since uniseriate rays are many in conifers, those that include radial resin ducts are multiseriate and named fusiform rays (IAWA Committee, 1964). The density of the radial ducts in tangential sections varies from 0.15 to 3.5 ducts per mm^2^ of secondary xylem (Wu and Hu, 1997). Axial and radial resin ducts occur in the secondary xylem of *Cathaya, Larix, Picea, Pinus* and *Psudotsuga* while *Keteleeria* has only vertical resin ducts (Wu and Hu, 1997; Esteban et al., 2021).

Traumatic resin ducts arise from cambial cells after trunk induction with metal pins in *Tsuga sieboldii* as documented by Kuroda and Shimaji (1983), and *Picea abies* trunk inoculated with *Ceratocystis polonica* (Nagy et al., 2000). They are also formed is hormone-mediated mode, it is assumed that both MJ (methyl jasmonate) and ethylene activate genes related to defense and formation of traumatic resin ducts (McKay et al., 2003; Schmidt et al., 2011). They appear relatively quickly after the injury, however, the time of year when the injury occurs is believed to be critical to the timing of traumatic resin duct onset (Gärtner and Heinrich, 2009).

Traumatic resin ducts are typically distributed in dense concentric series in the earlywood with a predominance in the vertical axis and occur singly or form an anastomosing network of cavities (Franceschi et al., 2002; Martin et al., 2002; Krokene et al., 2003). In *Cedrus*, are present in both vertical and radial systems in the vicinity of the wound (Fahn et al., 1979; Esteban et al., 2023). Traumatic resin ducts tend to be shorter than the resin ducts that are normal constituents of secondary xylem, however, in some cases they may be also longer and wider, scattered and found in remote areas from the injury (Krokene et al., 2008). Traumatic resin ducts are usually accompanied by small-diameter tracheids with thickened cell walls.

When considering the occurrence of resin ducts in compression wood, it should be noted that although Lee and Eom (1988) saw traumatic vertical resin ducts in *Pinus koraiensis* compression wood, this feature does not appear to be a consistent characteristic of compression wood. In species with resin ducts in secondary xylem, large areas with severe compression wood that fill the entire increment of secondary xylem often have no resin ducts, but in small areas of transient compression wood or in mild compression wood, resin ducts normally appear (Donaldson and Singh, 2013). In *Cedrus deodara*, Xu et al. (2018) found that branches with a 45° inclination and compression wood had more resin ducts than branches with a different inclination and other secondary xylem position.

Resin secretion is an important trait in the evolutionary adaptation of conifers to environmental conditions. It is chemically toxic, physically repels insects/pathogens and accounts for up to 1-5% of pine stem mass under normal growth conditions, but after treatment with chemical elicitors, an increase of resin content in the stem by 20% is observed (Stubbs et al., 1984; Wolter and Zinkel, 1984). *Pinus rigida*, *P. merkusii*, *P. ponderosa, P. caribaea* or *P. canariensis* have an extraordinary content of resin in the secondary xylem of trunk and are therefore referred to as pitch pines in commerce (Esteban et al., 2005). As pointed out by Hudgins et al. (2004), resin-producing members of the Pinaceae family are threatened by aggressive insects, while those with little or no resin have few or no aggressive pests. Despite the fact that plant defenses resulting from resin synthesis are costly and requires endogenous resource allocation and host energy input, they have been found to show trade-offs in growth, reproduction, and defensive traits (Herms and Mattson, 1992; Slack et al., 2017; Tuller et al., 2018; Watss et al., 2023). In addition, resin ducts as features of coniferous tree resistance may link dendrochronology and resin-based defense mechanisms (Slack et al., 2017; Zhao and Erbilgin, 2019; Hood et al., 2020; Vázquez-González et al., 2020; Catherwood et al., 2022).

It is also worth noting that in addition to the resin ducts in the sapwood, resin is also present in the heartwood, providing a defense against fungal decay and increasing the durability of the secondary xylem (Hillis, 1987; Piqueras et al., 2020; Bieniasz and Tulik, 2022). During formation of heartwood the resin ducts are frequently obstructed with tylosoids as a result of proliferation of the epithelial cells (Howard and Manwiller, 1969; Leggate et al., 2020) or due to the “fixation” of epithelial cells in a swollen state by events coincident with the formation of heartwood, including deposition of secondary cell walls and/or lignification as indicated by LaPascha and Wheeler (1990). Cown et al. (2011) found that a mature *Pinus radiata* stem contains approximately 25% heartwood with up to 10% resin content in the inner rings. Heartwood resin is stored in the lumen of the tracheids, but resinous tracheids may also be seen in sapwood which was reported in *Pinus elliottii* × *Pinus caribaea* by Leggate et al. (2019; 2020).

## 3 Resin-secreting cells in the secondary growth of monocotyledonous dragon trees

In the stem of the monocotyledonous dragon trees, secretion of red resin, called dragon’s blood, does not involve forming specialized structures (Fan et al., 2008; Jura-Morawiec and Tulik, 2015; Jura-Morawiec and Tulik, 2016). Resin-secreting parenchyma cells have no morphological features to distinguish them. It has been suggested that all of the living parenchyma cells in the secondary body of stem have the potential for resin secretion (Jura-Morawiec and Tulik, 2015), the only limitation is their lifespan. To date, the lifespan of these cells has not been investigated, but based on observations of lignin autofluorescence in the stem of *D. draco*, it has been concluded that it is related to the distance from the monocot cambium (Jura-Morawiec and Wiland-Szymańska, 2014).With increasing radial distance from the meristem, the cell walls of the parenchyma gradually become lignified, followed by cell death, which excludes them from resin production.

It is not clear how the resin is formed in parenchyma cells and transported along the tissue. Ding et al. (2020) have suggested that the major chemical constituents of dragon’s blood are transported out of the intracellular space in response to various stimuli by three potential transport mechanisms i.e., vesicle trafficking mediated transport, GST (glutathione S-transferase) transport or membrane transport. Recent studies of the leaf shedding of dragon trees have shown that dragon’s blood is in the form of vesicles, which have a tendency to aggregate and fill the cells or intercellular spaces (Jura-Morawiec et al., 2023). After the injury of the secondary tissues, resin typically fills the parenchyma cells and enters tracheids through the pits occluding their lumen (Cui et al., 2013; Jura-Morawiec and Tulik, 2015; Xu et al., 2022; **Figure 1B**). Resin secretion can be additionally enhanced by high humidity and fungal infection (Wang et al., 2010, 2011), and its accumulation increases after acid and sodium salt treatment (reviewed by Ding et al., 2020).

The mass of living parenchyma cells with the ability to secrete resin has a role in the dragon tree defense mechanism (Wang et al., 2010; Jura-Morawiec and Tulik, 2016). After the injury, the resin-filled parenchyma cells, together with the resin occluded lumen of the tracheids, limit the spread of infection/pathogen in all directions. In turn, the immediate solidification of the resin and its red aposematic (warning) color prevent access to living tissue, acting as a physical and chemical barrier. However, it should be noted that the appearance of the red resin color is a gradual process and is not visible immediately after the wound. In *D. draco*, it was observed two weeks after stem cutting (Jura-Morawiec and Tulik, 2015), while in *D. cochinchinensis* it was visible 3-4 days after wounding or fungal infection (Wang et al., 2011), with a clear red layer covering the wound site after 90 days (Xu et al., 2022).

## 4 Perspectives

In the course of evolution of the secondary growth, parenchyma cells have acquired various functions including resin synthesis and secretion. It seems that knowledge about the resin-based defense mechanisms of coniferous species may be useful in predicting the possibility of introducing alien coniferous species to new areas under the conditions of ongoing global warming. Undoubtedly, *Cedrus libani* or *P. canariensis* seem to be such species due to their high wound-healing capacity. In the case of monocotyledonous dragon trees, research efforts should focus on an in-depth understanding of their biology. Although this study has been going on since the 19th century, there is still a large gap in our understanding of dragon’s blood secretion, including the relationship between the lifespan of the parenchyma cells, the age of the dragon tree, and resin production. Analysis of the biological aging of parenchyma cells by measuring their metabolic activity in combination with histochemical techniques can help fill this knowledge gap.

## Author contributions

MT: Funding acquisition, Writing – original draft. JJ-M:Writing – review & editing and also prepared the microphotograph.
